# Inhibiting HMGB1 Reduces Cerebral Ischemia Reperfusion Injury in Diabetic Mice

**DOI:** 10.1007/s10753-016-0418-z

**Published:** 2016-09-05

**Authors:** Chong Wang, Jie Jiang, Xiuping Zhang, Linjie Song, Kai Sun, Ruxiang Xu

**Affiliations:** 1 The Military General Hospital of Beijing, PLA, Beijing, 100700 People’s Republic of China; 2 Jining First People Hospital, Jining, 272011 People’s Republic of China; 3 Jinan Central Hospital, Jinan, 250012 People’s Republic of China; 4 Graduate School, Weifang Medical University, Weifang, 261053 People’s Republic of China; 5 Affiliated Bayi Brain Hospital, General Hospital of Beijing, Military Region, No. 5, Nanmencang, Dongcheng District, Beijing, 100000 People’s Republic of China

**Keywords:** HMGB1, brain ischemia reperfusion, diabetes mellitus

## Abstract

High mobility group box1 (HMGB1) promotes inflammatory injury, and accumulating evidence suggests that it plays a key role in brain ischemia reperfusion (I/R), as well as the development of diabetes mellitus (DM). The purpose of this study was to investigate whether HMGB1 plays a role in brain I/R in a DM mouse model. Diabetes mellitus was induced by a high-calorie diet and streptozotocin treatment, and cerebral ischemia was induced by middle cerebral artery occlusion. We examined HMGB1 levels following cerebral I/R injury in DM and non-DM mice and evaluated the influence of altered HMGB1 levels on the severity of cerebral injury. Serum HMGB1 levels and the inflammatory factors IL-1β, IL-6, and inflammation-related enzyme iNOS were significantly elevated in DM mice with brain I/R compared with non-DM mice with brain I/R. Blocking HMGB1 function by intraperitoneal injection of anti-HMGB1 neutralizing antibodies reversed the inflammatory response and the extent of brain damage, suggesting that HMGB1 plays an important role in cerebral ischemic stroke in diabetic mice.

## INTRODUCTION

Cerebral ischemia reperfusion (I/R), causes 60–70 % of strokes [1] and leads to cerebral injury through a complex series of pathophysiological events, characterized by neuronal death and subsequent neurological dysfunction [2]. Despite intensive study, the mechanisms underlying brain inflammation remain poorly understood. Existing evidence suggests that HMGB1 plays a pivotal role in the pathogenesis of inflammation, which is a critical component of the cascade of events leading to the development of cerebral I/R [2–4].

High mobility group box1 (HMGB1) is a highly conserved non-histone DNA-binding nuclear protein that is ubiquitously expressed in eukaryotic cells and regulates transcription [5]. HMGB1 promotes inflammatory injury [6, 7] by inducing the expression of cytokines such as IL-1β, IL-6, and inflammation-related enzyme inducible nitric oxide synthase (iNOS) [4, 8–10], which are known to play key roles in the development of brain I/R [11–14]. The expression of HMGB1 is increased in diabetic rat models and diabetic patients [15–17] and evidence suggests that hyperglycemia may activate inflammatory signaling pathways [18, 19].

We investigated whether diabetes mellitus (DM) increases the severity of cerebral I/R by enhancing elevated HMGB1 expression after cerebral I/R injury. We established a mouse model of type 1 and type 2 DM that mimicked the physiological characteristics of DM in humans. Ischemia reperfusion injury was induced in our model by middle cerebral artery occlusion (MCAO) with minor modifications to the classic mouse model of diabetic stroke. By combining the diabetes and brain I/R models, the inflammatory reaction and relevant factors could be investigated. We examined the role of HMGB1 in the inflammatory response and severity of brain injury by blocking HMGB1 function with anti-HMGB1 neutralizing antibodies in diabetic stroke mice.

## MATERIALS AND METHODS

### Animals

Male C57BL/6 mice, 5–6 weeks of age, were housed in the Neurosurgical Research Center of Beijing Military Animal Center. All mice used in this study were handled according to the Center’s Health Guide for the Care and Use of Laboratory Animals.

### Treatment with HMGB1 Neutralizing Antibodies

The anti-HMGB1 polyclonal antibody (neutralizing antibody) and chicken IgY (isotype negative control antibody) were obtained from Tecan (Shanghai) Trading Co.,Ltd (Shanghai, China) [20]. Mice were injected intraperitoneally with 600 ug per mouse anti-HMGB1 polyclonal antibody or control IgY 1 h before ischemia as previously described [21].

### Mouse DM Model

Type 2 diabetes were induced in mice by a 3-week high-fat diet (DIO Rodent Purified Diet; TestDiet, Richmond, IN, USA, containing 61.6 % fat, 3140 Kcal), followed by an intraperitoneal (i.p.) injection with 20 mg/kg body weight streptozotocin (Sigma Aldrich, St. Louis, MO, USA) dissolved in saline [22, 23], then one additional week of high-fat feeding. Vehicle i.p. injections were administered to control mice in combination with a normal diet (LabDiet 5010, 5.5 % fat). Plasma glucose concentrations were measured using a blood glucose test meter in blood samples collected from the tail vein of mice at different time points (0, 2, 4, 6, and 8 weeks) after the induction of diabetes.

### Mouse Stroke Model

To achieve transient focal cerebral ischemia, we performed MCAO according to a modified intraluminal filament method as previously described [24]. In brief, mice were anesthetized by i.p. injection of ketamine and xylene, and a homeothermal blanket was used to maintain a rectal temperature of 36–37 °C. A vertical incision was made in the middle of the neck to expose the left common carotid artery, left internal carotid artery (ICA), left external carotid artery (ECA), and left pterygopalatine artery. The left ECA and the left pterygopalatine artery were ligated with a 5.0 silk suture and a small clip was used to occlude the left ICA at the bifurcation of the ICA and the pterygopalatine artery. A 6.0 nylon monofilament (0.2–0.22 mm) was inserted into the left ECA immediately after cutting with ophthalmic scissors. After removing the clip, the nylon monofilament was slowly pushed into the distal end of the middle cerebral artery. After 1 h of occlusion, the nylon thread was removed to initiate reperfusion. The arteries were surgically exposed but not occluded in the sham group. After 1 h of reperfusion, mice were sacrificed and brains were removed and processed for hematoxylin and eosin (H&E) staining and real-time PCR analysis.

### Blood Sample Collection

Blood samples were collected from the mice after experiments using the heart puncture procedure (without anticoagulants). Serum samples were collected by centrifugation of blood samples at 3000 rpm for 10 min.

### Fixation and Staining of Brain Tissue

Brain tissue was fixed in formaldehyde and embedded in paraffin wax. Five-micrometer slices were prepared and H&E staining was performed in the pathology lab at St. Michael’s hospital.

### Enzyme-Linked Immunosorbent Assay

Serum HMGB1 levels were measured using an enzyme-linked immunosorbent assay (ELISA) kit (R&D Systems). Briefly, 96-well ELISA plates were coated with HMGB1 mAbs (Cat. NO. ab12029; Abcam, Cambridge, MA, USA) and serum and standard protein samples were diluted 2–3 times in succession. Biotinylated antibodies were added followed by avidin-conjugated horseradish peroxidase. Tetramethylbenzadine substrate solution was added and the plate was read at 450 nm after 30 min. Serum HMGB1 concentrations were calculated from the standard curve.

### Evans Blue Extravasation

To determine changes in vascular permeability, a 4 % solution of Evans blue dye (Urchem, Shanghai, China) was injected intravenously into the tail vein of mice after reperfusion. Three hours later, mice were perfused with 150 ml of saline solution into the left ventricle of the heart and the brain was immediately removed and dissected into hemispheres. The weight of the left hemisphere was recorded. Fifty percent trichloroacetic acid was added to centrifuged brain tissues to extract the Evans blue dye. After centrifugation, the supernatant solution was diluted 1:3 in ethanol and the absorbance was determined at 620 nm. The concentration of Evans blue was calculated from a standard curve and the data were expressed as nanograms per gram of Evans blue.

### RNA Extraction and Real-Time PCR

Total RNA was extracted using Trizol reagent (Invitrogen Canada, Burlington, Canada) according to the manufacturer’s instructions. SuperScript II reverse transcriptase (Invitrogen Canada) was used to reverse transcribe the RNA and the PCR reaction was performed using the SYBR® green system (Applied Biosystems, Foster City, CA, USA). Reactions were monitored on an ABI Prism SDS 7000 (Thermo Scientific, Waltham, MA, USA) machine and results were analyzed with SDS 2.0 software.

The housekeeping gene *HPRT* (hypoxanthine-guanine phosphoribosyl transferase) was used as an internal control. Primers (Sigma-Aldrich) used were as follows: *HPRT* (accession number: NM_ J00423): left: 5′-caagcttgctggtgaaaagga-3′, right: 5′-tgaagtactcattatagtcaagggcatatc-3′; *IL-1β* (accession number: NM_ M15131): left: 5′-gtggaacttgaggccacatt-3′, right: 5′-tgtgacaaaaatgcctggaa-3′; *iNOS* (accession number: NM_ BC062378): left: 5′-caccttggagttcacccagt-3′, right: 5′-accactcgtacttgggatgc-3′, and *IL-6* (accession number: NM_ M24221): left: 5′-ccggagaggagacttcacag-3′, right: 5′-tccacgatttcccagagaac-3′, with the following primer cycling conditions: 95 °C for 15 s, 58 °C for 50 s, and 72 °C for 15 s (40 cycles).

### Statistical Analysis

Data analysis was performed using SPSS 13.0 (SPSS In, Chicago, IL, USA). Values are presented as mean ± standard deviation (SD). To compare between two different groups, a Student 2-tailed unpaired *t* test was applied. *p* values <0.05 were considered statistically significant (*p < 0.05, **p < 0.01, ***p < 0. 001, ****p < 0.0001).

## RESULTS

### Plasma Glucose Concentrations at Different Time Points

**Fig. 1:**
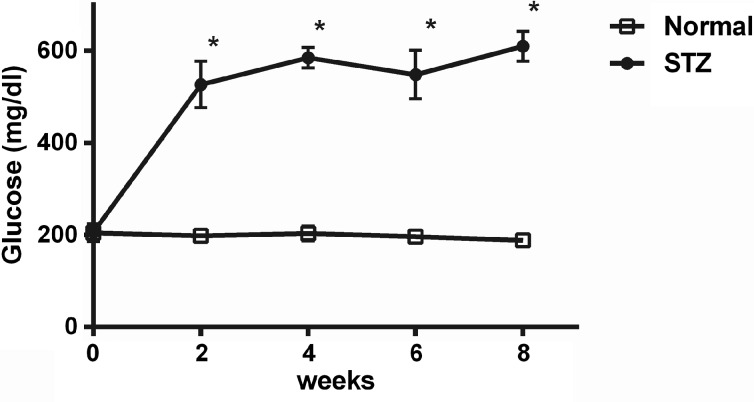
Blood glucose levels in the STZ group and control groups after glucose (or vehicle) administration. *p < 0.05.

Plasma glucose concentrations were measured 2, 4, 6, and 8 weeks after streptozotocin or vehicle injection in C57BL/6 J mice (Fig. 1). Streptozotocin treatment significantly increased the plasma glucose concentration at every investigated time point (*p* < 0.05).

### Effect of Transient Focal Cerebral Ischemia on Serum HMGB1 Levels

**Fig. 2:**
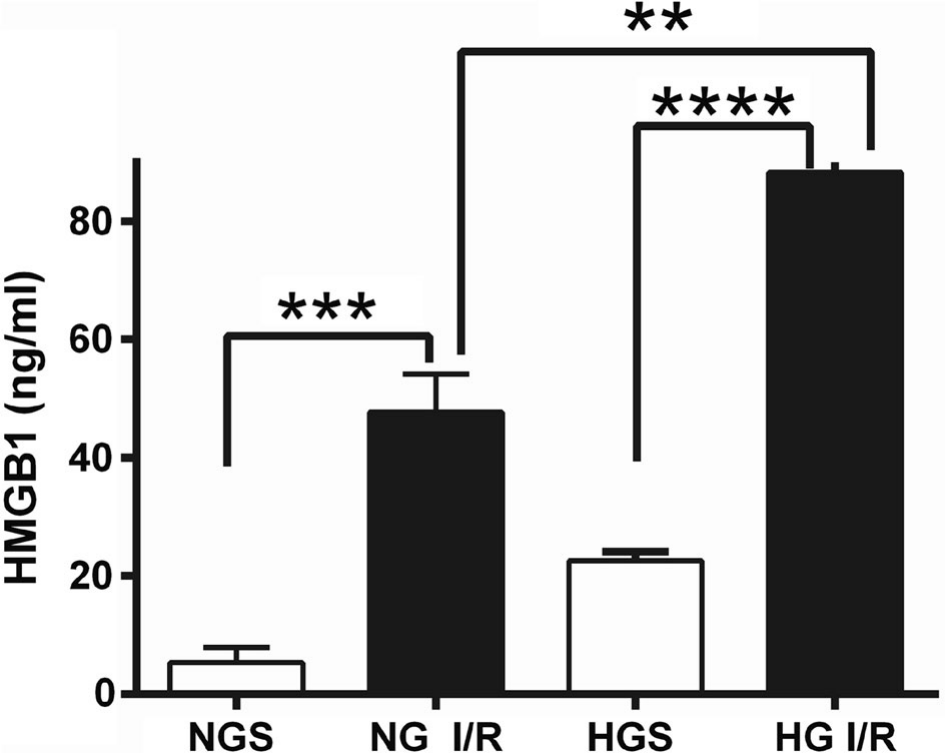
HMGB1 in serum of C57BL/6 J mice 1 h after reperfusion. Values were presented as mean ± SD, *n* = 5 for each group. **p < 0.01, ***p < 0.001, ****p < 0.0001. *NGS* normoglycemia sham, *NG I/R* normoglycemia ischemia/reperfusion, *HGS* hyperglycemia sham, *HG I/R* hyperglycemia ischemia/reperfusion.

The serum HMGB1 concentration was measured in normoglycemic sham (NGS), normoglycemic I/R (NG I/R), hyperglycemic sham (HGS), and hyperglycemic I/R (HG I/R) mice (Fig. 2). To test whether HMGB1 is involved in the pathology of cerebral I/R, we measured the HMGB1 concentrations 1 h after reperfusion in each group (*n* = 5). Serum HMGB1 levels in NG I/R group were significantly higher than the NGS group (*p* < 0.001) and serum HMGB1 levels in the HG I/R were significantly higher than the HGS group (*p* < 0.0001). Significantly higher serum HMGB1 levels were measured in hyperglycemic mice (both HG I/R and HGS groups) compared with sham mice (*p* < 0.01).

### Diabetes Aggravated Brain I/R Injury and Increased the Expression of IL-1β, IL-6, and iNOS

The increase of HMGB1 expression in diabetic stroke mice had a detrimental effect, as expected. To examine the severity of brain damage after diabetic stroke, we measured the extravasation of Evans blue dye and the expression of *IL-1β*, *IL-6*, and *iNOS* messenger RNA (mRNA) in all four groups (*n* = 5). Additionally, H&E staining was performed on brain sections to evaluate morphological changes. *IL-1β* and *iNOS* expression was significantly higher in the NG I/R group compared with the NGS group, however there was no difference in *IL-6* expression between these two groups (*p* > 0.05). *IL-1β* and *iNOS* expression was significantly elevated in HG I/R mice compared with HGS mice.

**Fig. 3:**
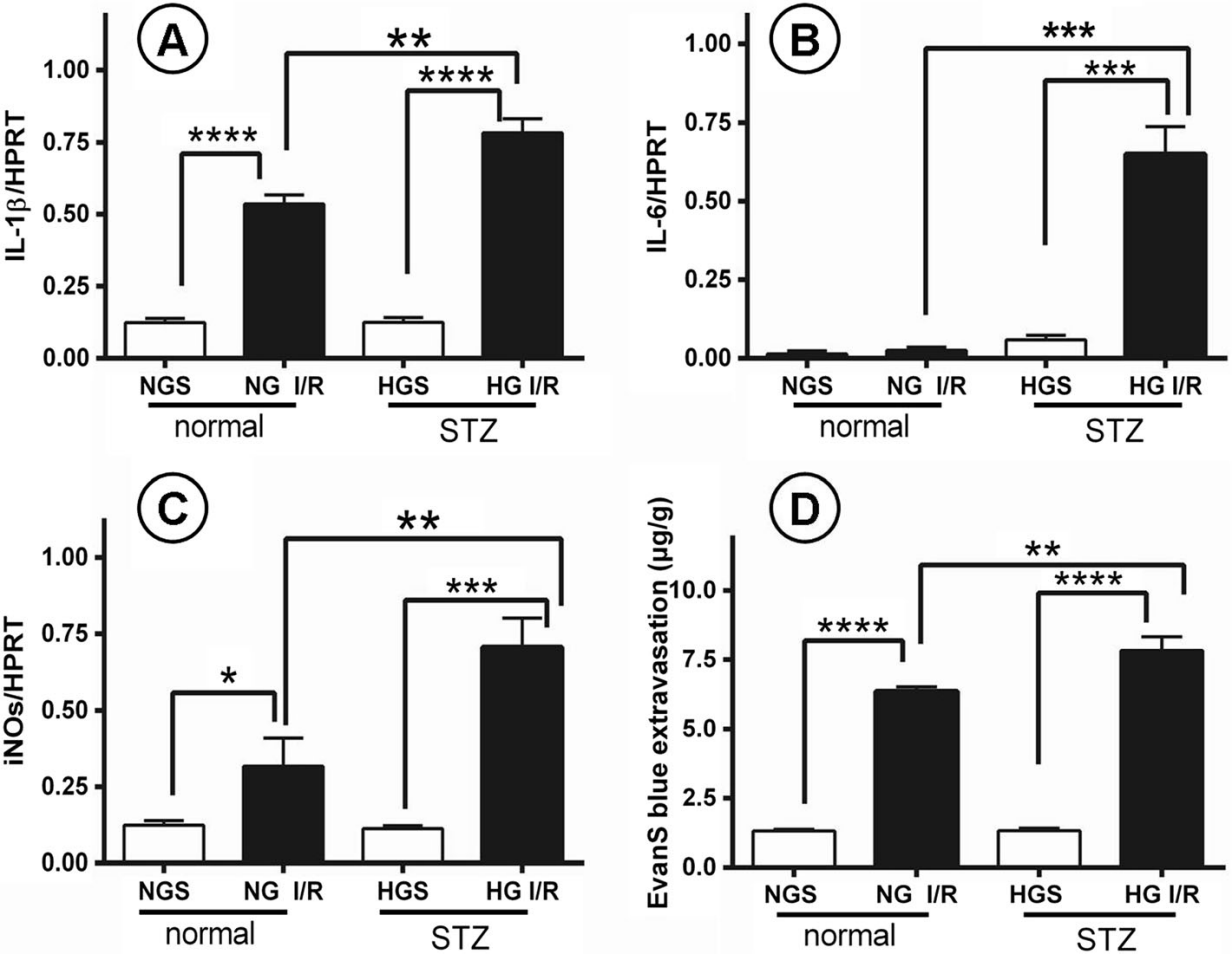
Brain I/R injury mice with DM aggravated brain injury and increased the expression of inflammation factors at the same time. **a**–**d** Analysis of blood–brain barrier damage and inflammation reaction in the process. **a**–**c** The expression of IL-1β, IL-6, and iNOS mRNA in four groups of brain tissues. **d** Evans blue extravasation, which represents for the breakdown of blood–brain barrier in each group. *n* = 5 for each group. Data are presented as mean ± SD, *p < 0.05, **p < 0.01, ***p < 0.001, ****p < 0.0001. *NGS* normoglycemia sham, *NG I/R* normoglycemia ischemia/reperfusion, *HGS* hyperglycemia sham, *HG I/R* hyperglycemia ischemia/reperfusion.

Expression of *IL-1β* (*p* < 0.0001), *IL-6* (*p* < 0.001), and *iNOS* (*p* < 0.001) was significantly elevated in the HG I/R group compared with the HGS group. Compared with the NG I/R group, *IL-1β*, *IL-6*, and *iNOS* expression was augmented in the HG I/R group (*p* < 0.01) (Fig. 3a–c). Evans blue extravasation measures the permeability of the blood–brain barrier (BBB) and revealed an increased breakdown of the BBB in hyperglycemic mice compared with normoglycemic mice (Fig. 3d). H&E showed more morphological brain damage in diabetic I/R group mice compared with non-diabetic I/R mice (Fig. 5a, c).

### Anti-HMGB1 Antibody Treatment Reduces Cerebral I/R Injury in Diabetic Mice

**Fig. 4:**
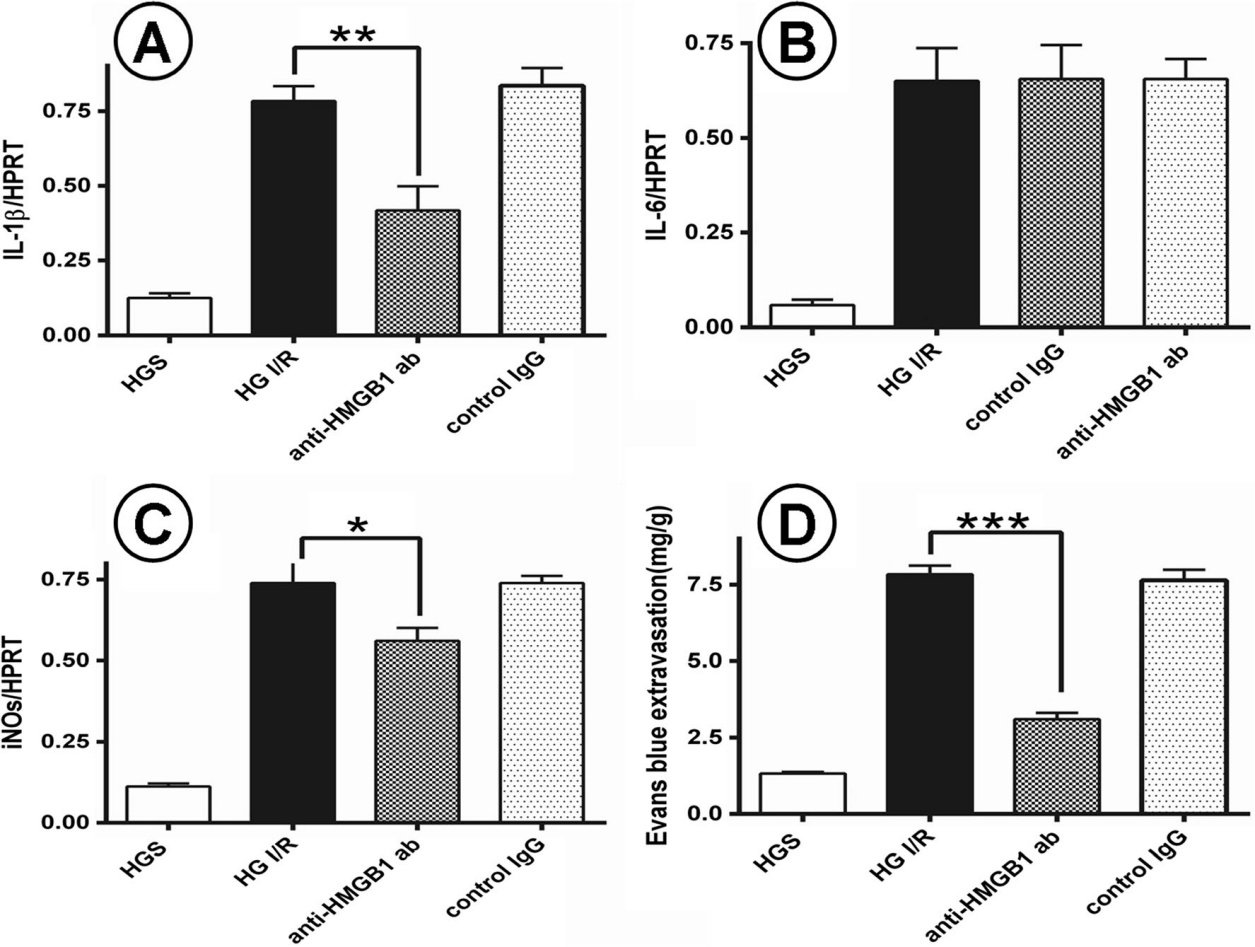
The protective role of anti-HMGB1 mAb in cerebral I/R injury with DM. **a**–**d** Analysis of blood–brain barrier damage and inflammation reaction in the process. **a**–**c** The expression of IL-1β, IL-6 and iNOS mRNA in 4 groups of brain tissues. **d** Evans blue extravasation, which represents for the breakdown of blood brain barrier in each group. *n* = 5 for each group. Data are presented as mean ± SD, *p < 0.05. *HGS* hyperglycemia sham, *HG I/R* hyperglycemia ischemia/reperfusion.

The augmented expression of HMGB1 after the onset of I/R implicates HMGB1 in the process of brain injury after I/R. To test the functional significance of HMGB1 release in our model, we injected anti-HMGB1 antibody to block HMGB1 function after brain injury. We observed that anti-HMGB1 antibody had a protective effect on cerebral I/R injury in DM mice (Fig. 4a–d), by reversing the negative impact of HMGB1 on cerebral I/R in diabetic mice.

#### Anti-HMGB1 Antibodies Reduce the Expression of IL-1, IL-6, and iNOS

It is well known that inflammatory cytokines, such as IL-1, IL-6, and inflammation-related enzyme iNOS, mediate I/R injury. To assess the anti-inflammatory effect of anti-HMGB1 antibody, we measured the expression of *IL-1β*, *IL-6*, and *iNOS*. Compared with the HGS group, *IL-1β*, *IL-6*, and *iNOS* expression was significantly higher in the HG I/R group (Fig. 4a–c). Treatment with anti-HMGB1 antibody markedly alleviated the inflammatory reaction by reducing the elevated expression of *IL-1β* (*p* < 0.01) and *iNOS* (*p* < 0.05) compared with the HG I/R group (Fig. 4a, c). However, anti-HMGB1 antibody treatment did not significantly affect *IL-6* expression (*p* > 0.05) (Fig. 4b).

#### Anti-HMGB1 Antibody Treatment and BBB Permeability

**Fig. 5:**
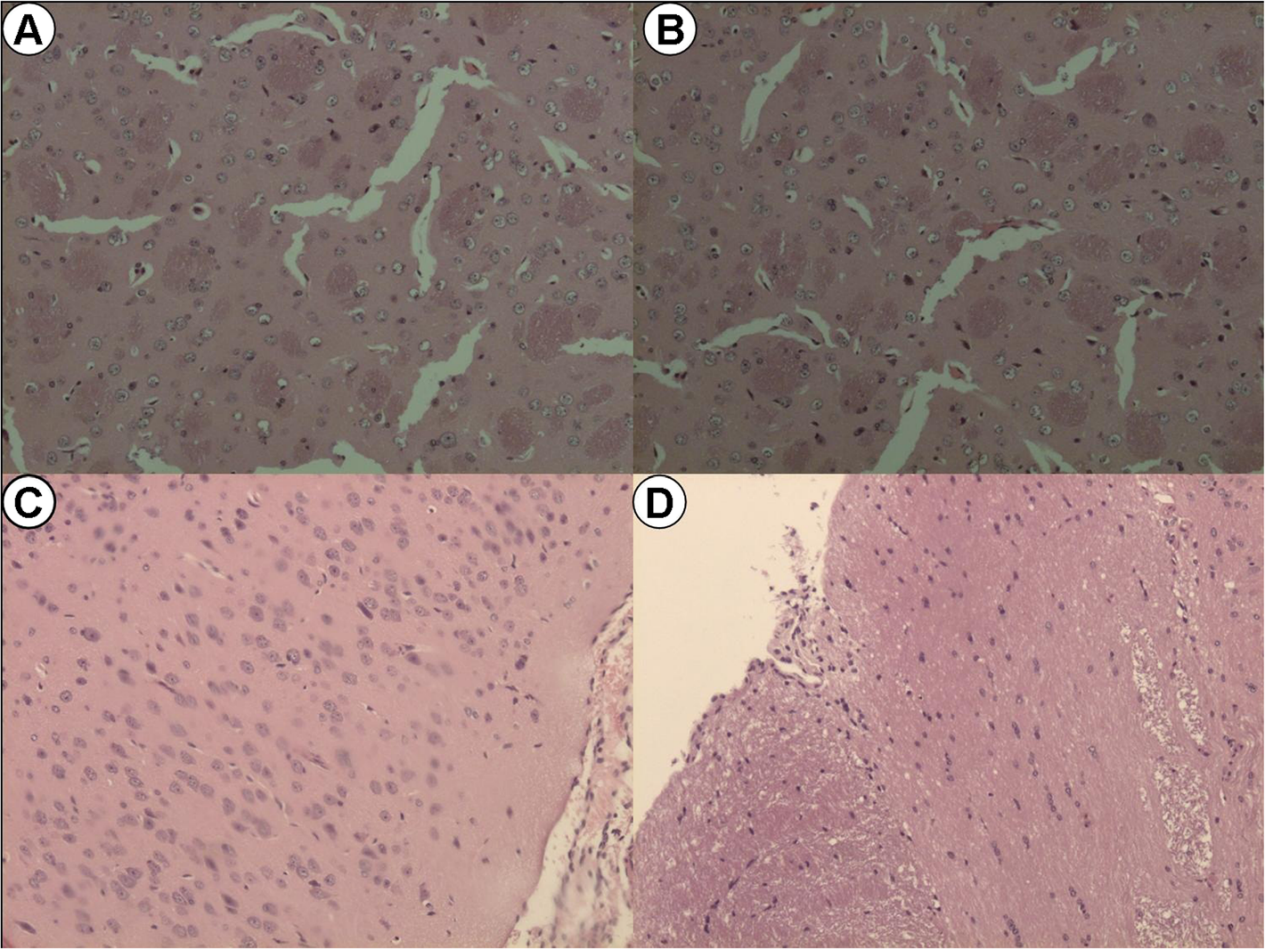
Representative images for the hematoxylin and eosin (H&E) staining in the formalin-fixed brain tissues (×100 magnification). **a** Hyperglycemia ischemia/reperfusion brain injury. **b** Hyperglycemia ischemia/reperfusion brain injury treated with anti-HMGB1 monoclonal antibody. **c** Normoglycemia sham group. **d** Hyperglycemia ischemia/reperfusion brain injury I/R treated with IgG.

The BBB is disrupted after cerebral I/R, which exacerbates brain injury. We used Evans blue extravasation to investigate BBB permeability after brain I/R injury. Increased Evans blue extravasation was observed after cerebral I/R injury in the HGS group (*p* < 0.001) (Fig. 4d), which was notably attenuated by anti-HMGB1 antibody treatment (Fig. 5b, d).

## DISCUSSION

In this study, we have shown that diabetes enhances the increase in HMGB1 serum levels and expression of the inflammatory cytokines IL-1β, IL-6, and inflammation-related enzyme after cerebral I/R injury in mice. We also confirmed that ischemic stroke leads to more severe brain injury in diabetic mice than non-diabetic mice, and that inflammation and brain injury can be ameliorated by blocking HMGB1 function with neutralizing antibodies. I/R injury is initiated by cell necrosis after prolonged ischemia and the inflammatory response is initiated by the restoration of blood flow to hypoxic tissue [25]. It is well-known that HMGB1 is passively released by necrotic cells and is able to activate leukocytes, which in turn secrete pro-inflammatory mediators, promoting further HMGB1 production [26].

In the present study, we discovered that levels of serum HMGB1 are significantly elevated after the onset of transient focal cerebral ischemia, consistent with previous findings that HMGB1 is released from neurons during the early stages of brain ischemic injury [25]. Kim *et al.* (2006) [2] also showed that concentrations of HMGB1 decreased in ischemic brain tissues, but increased in the serum. High levels of HMGB1 have been reported in the serum of patients who had suffered from stroke 7 days previously [27]. HMGB1 binds receptors, inducing signaling cascades that lead to an over-expression of pro-inflammatory molecules and cytokines [28]. In the current study, we identified IL-1β, IL-6, and iNOS as indicators of inflammation in the process of cerebral I/R, in agreement with previous findings [2, 29–31]. Breakdown of the BBB can induce brain edema and secondary brain injury in ischemic stroke and animals subjected to MCAO [32, 33]. In the present study, we examined the permeability of the BBB following cerebral I/R using Evans blue extravasation.

Diabetes mellitus is a metabolic disease that affects some vital organs, including the brain, in both microvascular and macrovascular ways [34]. Diabetes mellitus has been shown to increase the recurrence and adverse effects of ischemic stroke [35]. Both type 1 and type 2 DM are characterized by hyperglycemia, which induces inflammation and oxidative stress reactions [17, 36]. Therefore, the combination of DM and cerebral I/R increases the risk of more serious inflammation and brain injury. Hu *et al.*[37] showed that diabetic patients are two to four times more predisposed to stroke than patients without diabetes and are at a high risk of poor prognosis and mortality after stroke. This is supported by evidence from animal stroke models that hyperglycemia not only exacerbates stroke-related injury but also adversely affects the overall functional outcome [38].

In the current study, we have shown that HMGB1 levels were significantly higher in the DM cerebral I/R group compared with the DM sham group. This is in agreement with previous findings that diabetes promotes both the elevation of HMGB1 expression and a poor outcome after ischemic stroke in rats [39]. According to Li *et al.* (2013), hyperglycemia-mediated damage following ischemic stroke might include intracellular acidification, accumulation of reactive oxygen species, disruption of the BBB, and induction of the inflammatory response and axonal degradation [40]. In this study, we focused on the inflammatory response and BBB disruption following diabetic stroke; we found that expression of IL-1β, IL-6, and iNOS was strongly augmented in hyperglycemic mice, as was the permeability of the BBB. This is in agreement with previous findings that HMGB1 induces morphological and functional changes in the BBB, which can be inhibited by anti-HMGB1 antibodies [40].

We have confirmed that neutralizing anti-HMGB1 antibodies have a protective effect on the brain following MCAO in mice. Neutralizing HMGB1 antibodies have been shown to be a more effective treatment for brain I/R than the FK506 binding protein, matrix metalloproteinase inhibitors and radical scavengers [3]. The therapeutic effects of anti-HMGB1 antibodies on brain edema and BBB disruption induced by brain ischemia have been confirmed by Zhang *et al.* (2011).

In this study, we have provided evidence that treatment with neutralizing anti-HMGB1 antibodies significantly attenuates BBB disruption and morphological alterations induced by brain I/R injury in combination with DM; this suggests that anti-HMGB1 antibodies exert a protective effect on diabetic stroke injury. This is in agreement with previous findings that HMGB1 induces morphological and functional changes in the BBB, which can be inhibited by anti-HMGB1 antibodies [41].

Interestingly, we did not observe a decrease in the expression of IL-6 after treatment with anti-HMGB1 antibodies. This might reflect the undetermined role of IL-6 in brain I/R. IL-6 was recently shown to promote neurogenesis after ischemic stroke [42] and Joo Eun Jung *et al.* (2011) have reported a reduced brain infarct volume after injection of IL-6, suggesting a protective role for IL-6 in brain injury. However, over-expression of IL-6 may also induce the production of pernicious factors and contribute to the exacerbation of brain damage through inflammatory signaling cascades [43]. Therefore, IL-6 can exert both beneficial and detrimental effects on ischemic brain tissue. We observed an upregulation of IL-6 expression in the I/R injury group, that was not inhibited by HMGB1 neutralizing antibodies. This suggests a HMGB1-independent effect of IL-6 on the injured brain.

There are additional questions to be answered. First, it is not clear whether the elevated levels of HMGB1 in the serum are attributable to an increased inflammatory reaction, or several, critical mediators propagating cerebral I/R injury together with DM. Second, it is not known whether neutralizing HMGB1 antibodies alleviative cerebral injury by inhibiting inflammation activities or promoting protective effects. The mechanisms underlying the influence of HMGB1 in cerebral I/R injury together with DM remain to be determined.

In conclusion, we have provided evidence that the pathogenesis of DM increases HMGB1 expression, which aggravates cerebral I/R injury. The injection of neutralizing anti-HMGB1 antibodies alleviated brain injury and may represent a promising therapeutic approach for ischemic stroke in DM patients.

## Abbreviations

HMGB1: High mobility group box1
I/R: Ischemia reperfusion
DM: Diabetes mellitus
mAb: Monoclonal antibody
MCAO: Middle cerebral artery occlusion
CCA: Common carotid artery
ICA: Internal carotid artery
ECA: External carotid artery
ELISA: Enzyme-linked immunosorbent assay
BBB: Blood–brain barrier
